# COVID-19 vaccine acceptance and associated factors among health workers in West Guji zone, Southern Ethiopia: Cross-sectional study

**DOI:** 10.3389/fpubh.2023.974850

**Published:** 2023-02-10

**Authors:** Lechisa Asefa, Hailu Lemma, Chala Daba, Degefa Dhengesu, Mommedgezali Ibrahim

**Affiliations:** ^1^Department of Environmental Health, Institute of Health, Bule Hora University, Bule Hora, Ethiopia; ^2^Department of Environmental Health, College of Medicine and Health Sciences, Wollo University, Dessie, Ethiopia; ^3^Department of Environmental Health, College of Medicine and Health Sciences, Jimma University, Jimma, Ethiopia

**Keywords:** COVID-19, vaccine, acceptance, healthcare workers, Ethiopia

## Abstract

**Background:**

Currently, different COVID-19 vaccines are being developed and distributed worldwide to increase the proportion of the vaccinated people and as a result to halt the pandemic. However, the vaccination progress is different from place to place even among health care workers due to variation in vaccine acceptance. Therefore, this study aimed to assess the acceptance of COVID-19 vaccine and determinant factors among healthcare workers in west Guji zone, southern Ethiopia.

**Method and materials:**

An institutional-based cross-sectional study design was employed to assess COVID-19 vaccine acceptance and associated factors among health care workers from July to August 2021. A simple random sampling technique was used to choose 421 representative healthcare workers from three hospitals in the west Guji Zone. The self-administrated questionnaire was used to collect data. Bivariate and multivariable logistic regression analyses were computed to identify factors associated with the acceptance of the COVID-19 vaccine. *P* < 0.05 was considered for significantly associated factors.

**Result:**

From the representative health workers, 57, 47.02, and 57.9% of healthcare workers had good practice of COVID-19 prevention, adequate knowledge, and a positive attitude toward the COVID-19 vaccine consecutively. 38.1% of healthcare workers said they had a willingness to accept the COVI-19 vaccine. Profession (AOR-6, CI: 2.92–8.22), previous history of vaccine side effects (AOR: 3.67, CI: 2.75–11.41), positive attitude toward vaccine acceptance (AOR: 1.38, CI: 1.18–3.29), adequate knowledge toward COVID-19 vaccine (AOR: 3.33, CI: 1.36–8.12), and adequate practice of COVID-19 prevention measure (AOR: 3.45, CI: 1.39–8.61) were significant associated with COVID-19 vaccine acceptance.

**Conclusion:**

The proportion of COVID-19 vaccine acceptance among health workers was found to be low. From the study variables, profession, previous history of vaccine side effects, positive attitude toward vaccine acceptance, adequate knowledge to ward off COVID-19 vaccine, and adequate practice of COVID-19 prevention measures were significantly associated with COVID-19 vaccine acceptance.

## 1. Background

The COVID-19 pandemic has been a worldwide problem, with which all were challenged to control the spread of COVID-19 (1, 2). Over 235 million confirmed cases of SARS- CoV-2 infection as of October 2, 2021, with over 4.8 million documented fatalities, were found in 223 different country parts of the world (3). Since there were no known treatments for this pandemic, a number of strong interventions were used, including lockdowns, travel bans, isolation, closing schools and workplaces, limiting the size of gatherings, and the release of guidelines that included stringent public health measures like the wearing of masks, frequent hand washing, cleaning of surfaces, and social distancing policies (4, 5).

Healthcare professionals are at significant risk of contracting COVID-19 despite the fact that COVID-19 affects the entire community since they are frequently exposed to SARS-CoV-2 patients (6, 7). Protecting them from infection is crucial for their own health as well as the preservation of healthcare resources as it is estimated that at least 20% of healthcare professionals have the virus (6). Vaccines save millions of lives each year by preventing disease, disability, and death (8, 9). In order to boost vaccination uptake and create herd immunity to COVID-19, achieving high vaccination coverage among healthcare professionals helps to preserve the lives of health workers and also makes them role models for their family and patients (attendants) (10). Because of past influenza experiences, vaccination has been identified as the most effective way to stop outbreaks, lower morbidity, and death, particularly for healthcare workers (11).

Starting from an early time, vaccine hesitancy (VH) is an emerging public health challenge resulting from misinformation related to vaccine effectiveness and safety (12, 13). Immunization program success is being hindered by the rise of vaccine hesitancy, which is posing a threat to outbreaks of diseases that can be prevented by vaccination (12). One of the top 10 dangers to world health, according to WHO in 2019, is vaccination reluctance (14).

A study conducted in Vietnam on COVID-19 vaccine acceptance among health care workers showed 76.10% willingness to be vaccinated (7). Another study done in Ghana discovered that 39.3% of healthcare workers intended to receive the COVID-19 vaccine, and variables like sex, category of healthcare workers, relative having the disease, and confidence in the effectiveness of the government's COVID-19 prevention measures proved to be important predictors of vaccine acceptability (15).

COVID-19 vaccinations are being donated to developing nations like Ethiopia by a variety of donor nations in order to immunize high-risk populations like medical personnel and persons with chronic conditions (16). Even though Ethiopia is gaining vaccines, COVID-19 vaccine acceptance among health workers and the factors affecting it are not known. As with previous studies on COVID-19 vaccine acceptance, they have demonstrated that the acceptability of the vaccine differs depending on socio-demographic factors, such as race and educational level, as well as attitudes and beliefs regarding COVID-19 infection and vaccination (7, 17, 18). Responses in various nations indicate that acceptability of the COVID-19 vaccine has a significant degree of heterogeneity, according to a global survey that included 19 countries (19). As a result, it's critical to understand a vaccine is accepted in a given nation or region. In order to provide recommendations for ways to have a successful and seamless vaccination roll out plan for COVID-19, this study sought to explore the COVID-19 vaccine acceptance and associated factors among healthcare personnel.

## 2. Material and methods

### 2.1. Study area and period

This study was conducted in west Guji, which is located in southern Ethiopia. It is bordered on the south by the Borena zone; on the west by the Southern Nations, Nationalities, and Peoples Region; on the north by the Gedeo Zone of the Southern Nations, Nationalities, and Peoples Region and Sidama Region; and on the east by the Guji Zone. Its administrative center is Bule Hora Town. This zone has a total population of 1,424,267, of whom 105,443 are urban residents. The West Guji Zone had a total of three hospitals and 656 health workers.

### 2.2. Study design

The COVID-19 vaccine acceptance and associated factors were studied using an institutional-based cross-sectional study design.

### 2.3. Source population

The source population in this study was total health workers in west Guji zone public health hospitals.

### 2.4. Study population

Randomly selected representative health workers were the study population.

### 2.5. Study variables

#### 2.5.1. Dependent variable

COVID-19 vaccine acceptance.

#### 2.5.2. Independent variable

- Socio-demographic factor.
  ✓ Sex.
  ✓ Age.
  ✓ Marital status.
  ✓ Educational status.
  ✓ Profession.
  ✓ History of COVID-19 infection.
  ✓ Previous vaccination history.
  ✓ Family morbidity/death due to COVID-19.

- Knowledge of the workers.
- Attitude of workers.

### 2.6. Exclusion criteria

Worker on annual leave and severely ill during data collection.

### 2.7. Inclusion criteria

Nurses, medical doctors, midwife, medical laboratory workers, and other health workers were included in this study.

### 2.8. Sample size determination

The sample size was calculated using a single population proportion formula with a 95% confidence level assumption, a margin of error of 5%, and a 48.4% proportion of COVID-19 vaccine acceptance by health workers in south western Ethiopia (18) and a 10% non-response rate, yielding a final sample size of 421. Then the predetermined sample was proportionally allocated to three hospitals and a sample size of 211,114 and 96 health workers were drawn from Bule Hora Teaching Hospital, Melka Soda, and Kercha hospitals respectively.

### 2.9. Sampling technique and procedure

A simple random sampling technique was used to select study participants. The lists of total health workers, which serve as a frame of reference, were taken from hospitals' human resource offices. During data collection, the COVID-19 prevention measurement was implemented to minimize the risk of disease transmission. Questioners were distributed to the randomly selected health workers and fielded by them themselves (i.e., it was self-administrated).

### 2.10. A data collection tool

The structured self-administrated questionnaire was adapted after reviewing articles and guidelines (18, 20, 21). The questionnaire was prepared in English, translated to the local language, and then re-translated back to English to ensure consistency. The questionnaire contained eight parts, which were socio-demographic, health status and COVID-19 experience of health professionals, practice questions toward COVID-19, knowledge related factors of the respondents toward the COVID-19 vaccine, attitude toward the COVID-19 vaccine acceptance, and vaccine acceptance of health workers.

### 2.11. Quality control of data

Before actual data collection and revisions were completed, a pre-test was conducted on 5% of the sample of Yabelo hospital's medical staff. To guarantee the quality of the data, training was provided to data collectors and supervisors on the study's goal, its data collection methods, and its ethical considerations. The supervisors reviewed the accuracy and consistency of the data every day.

### 2.12. Data analysis

The data was checked, coded, and entered into Epi-data version 3.1 before being exported to SPSS version 26 for cleaning and analysis. Bivariate logistic regression was used to analyze the data and variables with a *p* ≤ 0.25 were selected for the multivariable logistic regression analysis. Multivariable logistic regression was computed to identify factors associated with vaccine acceptance among health workers. The variables with a *p* < 0.050 were taken as statistically significant associated with vaccine acceptance of health workers. The association was also presented with an AOR (adjusted odd ration) and a 95% confidence interval. Model fitness was checked using the Hosmer Lemeshow test.

### 2.13. Ethical clearance

Ethical clearance to undertake the study was obtained from the Bule Hora University Institute of Health Research and Community Service Directorate ethical review board. Informed consent was obtained from the chief clinical director of the hospitals and health workers after a brief explanation of the benefits of the study.

### 2.14. Operational definition

#### 2.14.1. Adequate knowledge

Knowledge scores above or equal to the mean score were assigned for adequate knowledge.

#### 2.14.2. Inadequate knowledge

Knowledge scores below the mean score were assigned for inadequate knowledge.

#### 2.14.3. Adequate practice

Practice scores above or equal to the mean score were assigned for adequate practice.

#### 2.14.4. Inadequate practice

Practice scores below the mean score were assigned for inadequate practice.

#### 2.14.5. Positive attitude

Attitude scores above or equal to the mean score were assigned for positive attitude.

#### 2.14.6. Negative attitude

Attitude scores below the mean score were assigned for negative attitude.

## 3. Results

### 3.1. Socio-demographic characteristics

A total of 421 self-administrated questions were returned with a response rate of 100%. The majority of the respondents were male (58.9%), aged > 30 (80.7%), married (55.4%), nurses (41.1%), degree holders (77.2%), and had an income of 91.3–182.4 dollars (33.2%) (Table 1).

**Table 1:** The socio-demographic characteristics of health workers of west Guji Zone, South Ethiopia 2021.

**S. no**	**Variable**		**Frequency**	**Percent**
1	Age	>30	340	80.7
		31–40	44	10.4
		41–59	37	8.9
2	Sex	Male	248	58.9
		Female	173	41.1
3	Marital status	Single	175	41.6
		Married	233	55.4
		Widowed	13	3.0
4	Profession	Nurse	173	41.1
		Physician (doctor)	44	10.4
		Midwifery	129	30.7
		Medical laboratory	21	5
		Pharmacy	54	12.9
5	Qualification	Diploma	90	21.3
		Degree	325	77.2
		Masters	6	1.5
6	Income in dollar ($)	68.4	123	29.2
		91.2	94	22.3
		91.3–182.4	140	33.2
		>182.4	64	15.3
7	Use of broadcast media	Yes	311	73.8
		No	110	26.2
8	Trained on COVID-19	Yes	184	43.6
		No	237	56.4

### 3.2. The health status and COVID-19 experience of healthcare professionals

Of the total participants, 93.5% of them have no personal history of COVID-19. Of the health workers who have tested for COVID-19, 21.7% of the health workers tested positive for the virus. 73.4% of health workers have previously received any type of vaccine and in 38.1% of them, vaccine side effects were manifested (Table 2).

**Table 2:** The health status and COVID-19 experience of health the professionals 2021.

**S. no**	**Variable**		**Frequency**	**Percent (%)**
1	Personal history of COVID-19 infection	Yes	27	6.5
		No	394	93.5
2	Know any friends, neighbors, or colleagues infected by Coronavirus	Yes	171	40.6
		No	250	59.4
3	Have tested for COVID-19	Yes	125	29.7
		No	296	70.3
4	Result of COVID-19 test	Positive	27	21.7
		Negative	98	78.3
5	Heard about the COVID-19 vaccine	Yes	329	78.2
		No	92	21.8
6	Do you have any of the chronic disease	Yes	27	6.4
		No	394	93.6
7	Have receive any type of vaccine previously	Yes	313	74.3
		No	108	25.7
8	If Question no. 7 is “yes” is there any vaccine side effect that was manifested on you?	Yes	119	38.1
		No	194	61.9

### 3.3. Healthcare workers' COVID-19 prevention practices

Of total health workers, 57% of them had good practice of COVID-19 prevention measures. The majority (91.1%) had washed their hands regularly or frequently. However, 56.9 % of the workers didn't cover their cough and sneeze with tissue/handkerchief (Table 3).

**Table 3:** The COVID-19 prevention practice of health workers 2021.

**S. no**	**Variable**		**Frequency**	**Percent**
1	Did the outbreak of the COVID-19 virus make you increase the frequency of washing hands?	Yes	384	91.1
		No	37	8.9
2	Did you carry hand sanitizer with you during the outbreak in Ethiopia?	Yes	358	85.1
		No	63	14.9
3	Did you write down or store in your phone any helpline number to contact in case you suspected that you or someone you know has the COVID-19 virus?	Yes	219	52.0
		No	202	48.0
4	Did you maintain social distance during the outbreak?	Yes	365	86.6
		No	56	13.4
5	Did you cover coughs and sneeze with a tissue/handkerchief during the outbreak?	Yes	181	43.1
		No	240	56.9
6	Did you avoid unnecessary travel or outing during the outbreak?	Yes	189	45
		No	232	55
7	Did you dispose used mask in dust bin?	Yes	373	88.6
		No	48	11.4
8	Do you wash your hands after sneezing or coughing?	Yes	210	49.9
		No	169	40.1
9	Do you touch your face, nose, or mouth with your unclean hands?	Yes	184	43.6
		No	237	56.4
10	In order to prevent contracting and spreading COVID-19 I avoid handshaking, hugging and kissing	Yes	367	87.1
		No	54	12.9

### 3.4. Knowledge of the respondents toward the COVID-19 vaccine

Among the health workers, only 47.02% have good knowledge of the COVID-19 vaccine. 77.2% of the health profession responded that COVID-19 was not completely safe and 19.3% of the workers didn't know that the COVID-19 vaccine started in Ethiopia (Table 4).

**Table 4:** Knowledge of the respondents toward COVID-19 vaccine.

**S. no**	**Variable**		**Frequency**	**Percent**
1	Vaccine will help to provide long term immunity	Yes	384	91.1
		No	37	8.9
2	Vaccine helps to reduce risk of virus infection	Yes	400	95.0
		No	21	5.0
3	AstraZeneca and Covishield are the two vaccines used in Ethiopian	Yes	311	73.8
		No	110	26.2
4	Vaccination is an effective way to prevent and control COVID-19	Yes	117	27.7
		No	304	72.3
5	COVID-19 is affect more elder than young people	Yes	386	91.6
		No	35	8.4
6	COVID-19 vaccine is completely safe	Yes	96	22.8
		No	325	77.2
7	The vaccine of COVID-19 has started in Ethiopia	Yes	340	80.7
		No	81	19.3
8	Do you have a high risk of COVID-19 transmission at work	Yes	333	79.2
		No	88	20.8

### 3.5. Attitude toward the COVID-19 vaccine acceptance

From total health workers, 57.9% had a positive attitude toward the COVID-19 vaccine. Only 43.6% of health workers believed the COVID-19 vaccine was necessary to prevent COVID-19 and 93.1% of health workers believed the COVID-19 vaccine had side effects (Table 5).

**Table 5:** Attitude toward the COVID-19 vaccine acceptance.

**S. no**	**Variable**		**Frequency**	**Percent**
1	Do you have trust on COVID-19 vaccine	Yes	271	64.4
		No	150	35.6
2	Do you believe that COVID-19 vaccine has side effect	Yes	392	93.1
		No	29	6.9
3	Do you believe that taking COVID-19 vaccine can contradict with your religion	Yes	125	29.7
		No	296	70.3
4	Do you think you are susceptible to the infection of COVID-19 diseases	Yes	263	62.4
		No	158	37.6
5	Do you believe that the vaccine is necessary for the prevention of COVID-19	Yes	184	43.6
		No	237	56.4
6	It is not possible to reduce the incidence of COVID-19 without vaccination	Yes	325	77.2
		No	96	22.8

### 3.6. Acceptance of the COVID-19 vaccine

Among the study participants, 61.9% didn't have the willingness to receive the COVID-19 vaccine and the major reasons for not accepting the vaccine were fear of side effects (34%) (Table 6).

**Table 6:** COVID-19 vaccine acceptance.

**S. no**	**Variable**		**Frequency**	**Percent**
1	Are you willingness to accept COVID-19 vaccine if it will available for you?	Yes	160	38.1
		No	261	61.9
2	If question above was No. what the reason?	Inadequate data about the safety of the vaccine	56	21.3
		Fear of adverse effects of the vaccine	89	34
		Vaccine causing COVID-19	4	1.5
		I prefer other ways of protection	18	7
		Prior adverse reaction to any vaccine	55	21
		Religion issue	40	15.2

### 3.7. Factors associated with COVID-19 vaccine acceptance

Multivariate analysis reveals that healthcare workers with a physician profession were 6 times more likely to accept the COVID-19 vaccine. Health workers who have adequate COVID-19 prevention practice were 3.45 times more likely to accept the COVID-19 vaccine than the rest (Table 7).

**Table 7:** Factor associated with COVID-19 vaccine acceptance.

**Variable**	**Univariate analysis**		**Multivariate logistic regression**	
	**COR (CI)**	***P*** **-value**	**AOR (CI)**	***P*** **-value**
**Profession**				
Nurse	1	1	1	
Doctor	5.6 (3.03–7.91)	<0.001	6 (2.92–8.22)^*^	<0.001
Pharmacy	1.08 (0.55–12.75)	0.78	0.14 (0.01–2.83)	0.89
Midwifery	1.92 (0.60–5.35)	0.26	1.76 (1.97–2.18)	0.23
Medical laboratory	0.07 (0.02–9.55)	0.98	1.19 (0.78–3.76)	0.43
**Marital status**				
Single	1	1	1	
Married	2.14 (0.03–3.78)	0.54	1.22 (1.67–5.24)	0.114
Widowed	1.12 (0.45–2.87)	0.512	1.33 (0.026–0.67)	0.322
Divorced	0.89 (0.12–2.76)	0.887	0.18 (0.372–3.136)	0.97
Any vaccine side effect that was manifested previously (yes/no)	2.15 (1.196–3.858)	0.010	3.67(2.75–11.41)^*^	0.000
Sex (male vs. female)	0.529 (0.297–0.944)	0.31	1.23 (0.76–5.7)	0.13
Training on vaccine (yes/no)	2.93 (1.630–5.277)	0.012	1.49 (0.32–9.19)	0.071
Know any friends, neighbors, or colleagues infected by Coronavirus (yes/no)	1.95 (1.096–3.494)	0.023	0.99 (0.31–3.25)	0.993
Use of medias (yes/no)	0.540 (0.286–1.021)	0.158	0.245 (0.071–0.84)	0.56
Knowledge of vaccine (Adequate vs. Inadequate)	2.87 (1.592–5.181)	0.000	3.33 (1.36–8.12)^*^	0.008
Attitude toward vaccine (positive vs. negative)	2.031 (1.138–3.625)	0.017	1.38 (1.18–3.29)^*^	0.0369
Practice of other COVID-19 prevention measure (adequate vs. Inadequate)	2.37 (1.32–4.26)	0.004	3.46 (1.39–8.61)^*^	0.008

^*^Shows variable significant, COR, Crude odd ratio; AOR, Adjusted odd ratio; CI, Confidence interval.

## 4. Discussion

Vaccines are one of the most important means of disease prevention during a pandemic (22). The effectiveness of vaccination is determined by the acceptance of vaccines by the community (23). The recent studies focused on the COVID-19 vaccine acceptance by healthcare workers and associated factors. The response rate in this study was 100%, probably healthcare workers have an attitude to take part in survey or cross sectional study and response rate is higher than the one reported by similar studies (ranging from 63 to 90%) (24). One of the key elements influencing health care workers' intentions to obtain the COVID-19 immunization is knowledge. The findings of this study reveal that only 47.02% of the health workers had adequate knowledge about the COVID-19 vaccine. This finding was lower than studies conducted on health workers located in south western Ethiopia (16) and Pakistan (22). This discrepancy may be explained by variations in study environments, study times, and the involvement of regulatory bodies in the dissemination of COVID-19 vaccination knowledge.

In this finding, the knowledge of health workers toward vaccines was associated with COVID-19 vaccine acceptance (AOR = 3.33, 95% CI: 1.366–8.112) and it indicates improving awareness of health workers is necessary to increase COVID-19 vaccine acceptance in Ethiopia. This study was similar to a study conducted in Vietnam which found that people who had good knowledge were 3.37 times more likely to have vaccine acceptance (AOR = 3.37; 95% CI: 1.04–10.86, *P* < 0.05) (7).

Furthermore, this study also reveals that 57% of the health workers had adequate COVID-19 prevention practice but, specifically, 56.9% of the workers didn't use tissue/handkerchief to cover their cough and sneeze. Workers' practice was also associated with vaccine acceptance; those who had adequate practice for COVID-19 prevention measures were more likely to accept vaccine when it became available to them (AOR = 2.37, 95% CI: 1.32–4.26, *P* = 0.004).

Regarding the attitude of the workers toward vaccines, 57.9% of health workers have a positive attitude and 43.6% of health workers believe the COVID-19 vaccine is necessary to prevent COVID-19. This study was lower than a study conducted in south western Ethiopia, which found 65.6% of workers have a positive attitude (18). A recent study found that vaccine acceptance has a significant association with having a positive attitude toward the vaccine (AOR, 2.031, 95% CI: 1.138–3.625, *p* = 0.017) and it was comparable with other studies (21, 23).

Moreover, 38.1% of the health workers had a willingness to accept the vaccine, which was lower than expected since willingness to accept COVID-19 was expected to be high among health workers. This study finding was lower than studies conducted in Vietnam (7), French (17), and Iran (25) which recorded 76,76.9, and 62.1% of healthcare providers would accept a vaccine, respectively. However, it was higher than the studies done in Congo and Hong Kong, ranging from 27.7 to 40% (20, 24). Acceptance of health workers is related to educational status, profession, previous vaccine side effects, knowledge, and attitude. This association was supported by other studies conducted in Ethiopia (4, 12), which have found associations between vaccine acceptance and profession, attitude and preventive practice; and Vietnam, which have found associations between vaccine acceptance and profession, use of media, knowledge, and belief (7).

According to the results of the current study, doctors were nearly six times more likely than other health professionals to be willing to receive the COVID-19 vaccine. This result was consistent with another study that found that doctors were more likely than other health workers to accept the COVID-19 immunization (12, 19).

This study has limitations since it was a cross-sectional study and it was done only on health workers in west Guji zone hospitals; it did not include private health organizations or health workers in health centers and other government institutions. However, this finding has come up with concrete data about the vaccine acceptance of health workers in the west Guji Zone.

## 5. Conclusion

In conclusion, the vaccine acceptance rate (38.1%) of the health works was low. From the study variables, profession, previous history of vaccine side effects, positive attitude toward vaccine acceptance, adequate knowledge to ward off COVID-19 vaccine, and adequate practice of COVID-19 prevention measures were significantly associated with COVID-19 vaccine acceptance. The emphasis should be given for health care workers and the awareness creation should be done special on vaccine safety.

## Data availability statement

The original contributions presented in the study are included in the article/Supplementary material, further inquiries can be directed to the corresponding author.

## Ethics statement

The studies involving human participants were reviewed and approved by Fistum Demisse. The Ethics Committee waived the requirement of written informed consent for participation.

## Author contributions

LA contributed to designing the study, analyzed the data, interpreted the results, and performed the manuscript drafting. DD, HL, and CD contributed to the results interpretation and manuscript drafting. All authors confirmed and approved the final version for submission.
