# Supplementary material for: COVID-19 vaccine acceptance and associated factors among health workers in West Guji zone, Southern Ethiopia: Cross-sectional study
Source: Front Public Health. 2023 Feb 10;11:974850. doi: 10.3389/fpubh.2023.974850 (PMC9950729; doi:10.3389/fpubh.2023.974850)
Supplement: Supplementary file 1 [file Data_Sheet_1.PDF]

COVID-19 vaccine acceptance and associated factors among health workers in West Guji zone southern, Ethiopia: Cross-sectional study

Lechisa Asefa[1*] , Hailu Lemma[1], Chala Daba[2], Degefa Dhengesu[1], Mommedgezali Ibrahim[3]

[1]Deparrtment of Environmental Health, Institute of Health, Bule Hora University, Ethiopia

[2]Departement of environmental health, College of Medicine and Health Sciences, Wollo University, Ethiopia

[3]Department of environmental health, College of Medicine and Health Sciences, Jimma University, Jimma, Ethiopia

Corresponding Author

Lechisa Asefa: - Email of correspondence: - Lechisa123@gmail.com

Appendix 1:-questioner to assess the COVID-19 vaccine acceptance and associated factors among health workers in West Guji zone from July–August 2021.

Questioners ID--------------------

Name of data collector………………signature…………….Date----/----/2021

Name of supervisor ………………signature…………….Date----/----/2021

Part one. The socio-demographic characteristics of health workers in West Guji zone southern, Ethiopia

S.no	Question	Answer	Remark
1	Age?	>30	
		31-40	
		41-59	
2	Sex?	Male	
		Female	
3	Marital status?	Single	
		Married	
		Widowed	
4	Profession?	Nurse	
		Physician(doctor)	
		Midwifery	
		Medical laboratory	
		Pharmacy	
5	Qualification?	Diploma	
		Degree	
		Masters	
6	Income in Dolar($)?	68.4	
		91.2	
		91.3-182.4	
		>182.4	
7	Use of broadcast media?	Yes	
		No	
8	Trained on COVID-19?	Yes	
		No	

Part two. SARS- CoV-2 infection experience of health the professionals of health workers in West Guji zone southern, Ethiopia

S.no	Variable		Remark
1	Personal history of COVID-19 infection	Yes	
		No	
2	Know any friends, neighbors, or colleagues infected by Coronavirus	Yes	
		No	

3	Have tested for COVID-19	Yes	
		No	
4	Result of COVID-19 test	Positive	
		Negative	
5	Heard about the COVID-19 vaccine	Yes	
		No	
6	Do you have any of the chronic disease	Yes	
		No	
7	Have receive any type of vaccine previously	Yes	
		No	
8	If Question no. 7 is ‘‘yes’’ is there any vaccine side effect that was manifested on you?	Yes	
		No	

Part three. The COVID-19 prevention practice of health workers in West Guji zone southern, Ethiopia

S.no	Variable		Remark
1	Did the outbreak of the COVID-19 virus make you increase the frequency of washing hands?	Yes	
		No	
2	Did you carry hand sanitizer with you during the outbreak in Ethiopia?	Yes	
		No	
3	Did you write down or store in your phone any helpline number to contact in case you suspected that you or someone you know has the COVID-19 virus?	Yes	
		No	
4	Did you maintain social distance during the outbreak?	Yes	
		No	
5	Did you cover coughs and sneeze with a tissue / handkerchief during the outbreak?	Yes	
		No	
6	Did you avoid unnecessary travel or outing during the outbreak?	Yes	
		No	
7	Did you dispose used mask in dust bin?	Yes	
		No	
8	Do you wash your hands after sneezing or coughing?	Yes	
		No	
9	Do you touch your face, nose, or mouth with your unclean hands?	Yes	
		No	
10	In order to prevent contracting and spreading COVID-19 I avoid handshaking, hugging and kissing	Yes	
		No	

Part four. Knowledge of health workers towards COVID-19 vaccine in West Guji zone southern, Ethiopia

S.no	Variable		Remark
1	Vaccine will help to provide long term immunity	Yes	
		No	
2	Vaccine helps to reduce risk of virus infection	Yes	
		No	
3	AstraZeneca and Covishield are the two vaccines used in Ethiopian	Yes	
		No	
4	Vaccination is an effective way to prevent and control COVID-19	Yes	
		No	
5	COVID-19 is affect more elder than young people	Yes	
		No	
6	COVID-19 vaccine is completely safe	Yes	
		No	
7	The vaccine of COVID-19 has started in Ethiopia	Yes	
		No	
8	Do you have a high risk of COVID-19 transmission at work	Yes	
		No	

Part Five. Attitude towards the COVID-19 vaccine acceptance among health care workers in west Guji southern, Ethiopia

S.no	Variable		Remark
1	Do you have trust on COVID-19 vaccine	Yes	
		No	
2	Do you believe that COVID-19 vaccine has side effect	Yes	
		No	
3	Do you believe that taking COVID-19 vaccine can contradict with your religion	Yes	
		No	
4	Do you think you are susceptible to the infection of COVID-19 diseases	Yes	
		No	
5	Do you believe that the vaccine is necessary for the prevention of COVID-19	Yes	
		No	
6	It is not possible to reduce the incidence of COVID-19 without vaccination	Yes	
		No	

Part six. COVID-19 vaccine acceptance of health workers in West Guji zone southern, Ethiopia

s.no	Variable		Remark
1	Are you willingness to accept Covid-19 vaccine if it will available for you?	Yes	
		No	
2	If question above was No. what the reason?	Inadequate data about the safety of the vaccine	
		Fear of adverse effects of the vaccine	
		Vaccine causing COVID-19	
		I prefer other ways of protection	
		Prior adverse reaction to any vaccine	
		Religion issue	
